# Effects of Frozen Storage Temperature on Water-Holding Capacity and Physicochemical Properties of Muscles in Different Parts of Bluefin Tuna

**DOI:** 10.3390/foods11152315

**Published:** 2022-08-03

**Authors:** Jinfeng Wang, Hanwen Zhang, Jing Xie, Wenhui Yu, Yuyao Sun

**Affiliations:** 1 College of Food Science and Technology, Shanghai Ocean University, Shanghai 201306, China; jfwang@shou.edu.cn (J.W.); zhw1045993510@163.com (H.Z.); strfor@163.com (W.Y.); bj2012_0225@126.com (Y.S.); 2 Shanghai Professional Technology Service Platform on Cold Chain Equipment Performance and Energy Saving Evaluation, Shanghai 201306, China; 3 Shanghai Engineering Research Center of Aquatic Product Processing & Preservation, Shanghai 201306, China; 4 National Experimental Teaching Demonstration Center for Food Science and Engineering, Shanghai Ocean University, Shanghai 201306, China

**Keywords:** bluefin tuna, frozen storage, physicochemical properties, water-holding capacity, color difference

## Abstract

The effects of different freezing temperatures on the water-holding capacity and physicochemical properties of bluefin tuna were studied. The naked body, big belly and middle belly parts of bluefin tuna were stored at −18 °C and −55 °C for 180 days. The tuna was evaluated by determining the water-holding capacity, color difference, malondialdehyde (MDA), salt-soluble protein content, free amino acid (FAA), endogenous fluorescent proteins and water distribution and migration. The salt-soluble protein content was measured by the Bradford method. The color difference was measured by a CR-400 color difference meter. The water distribution and migration were analyzed by the low-field nuclear magnetic resonance (LF-NMR). The results showed little quality change during short-term frozen storage, but the frozen storage temperature of −55 °C significantly improved the quality of tuna compared with the frozen storage temperature of −18 °C. There were great differences in the salt-soluble protein content, water-holding capacity and water content the different parts of the tuna. The water-holding capacity and the protein content were the highest, and the water distribution of the naked body part was the most uniform of the three different parts. Because of the high fat content in the big belly and the middle belly, the MDA content and the odor of amino acid increased rapidly and the quality seriously decreased during the frozen storage.

## 1. Introduction

As is well-known, tuna is highly perishable, and therefore, storage time can influence the quality of tuna. Generally, frozen storage is chosen as the main technique for extending the shelf life of tuna [1]. Compared with ice storage and cold storage, frozen storage uses a lower storage temperature. The effect of beetroot-peel dip treatment on steak [2] was studied by Hafsa et al. who found that cellular enzymatic activity and biochemical reactions were more inhibited when the frozen storage was at a relatively low temperature. Similarly, frozen storage was found to be an essential technology in meat processing for a long shelf life and good quality [3].

Tuna is a good source of nutrients such as protein, fat, vitamins, amino acids, unsaturated fatty acids, and minerals [4]. Bluefin tuna is suitable as a raw material for the preparation of high quality food [5]. Based on their research on the effects of ultra-low temperature and freshness on the quality of frozen tuna meat, Naho et al. [6] found that the frozen storage temperature of tuna should be −50 °C or lower. During the freezing process, ice crystals are formed in muscle cells, and Teuteberg et al. [7] investigated the effects of frozen storage time and temperature on the quality of thawed pork and found that ice crystals can destroy muscle cells and change the cell structure, resulting in water loss and affecting the meat quality. The water-holding capacity is affected by the ice crystals [8]. For bluefin tuna, different parts contain different nutrients [9]. Freezing different parts of the tuna can maximize its economic value.

The quality of frozen storage products may be affected by several factors, such as the frozen storage mode [10], frozen storage temperature [11], freezing rate [12], etc. According to the research, the frozen storage temperature has a significant impact on the quality of frozen tuna [13], and the water-holding capacity is an important quality parameter that characterizes the sensory characteristics of tuna, thus affecting the quality of tuna.

However, there are few studies on the impact of frozen storage temperature on bluefin tuna’s physicochemical properties, as well as the water-holding capacity. There is no public research on the frozen storage of different parts of bluefin tuna. Research on the frozen storage of different parts will help to improve the economic benefits of bluefin tuna. Therefore, the purpose of this study was to explore the effects of different frozen storage temperatures on the water-holding capacity and physicochemical properties of bluefin tuna and to provide a theoretical foundation for improving the fresh-keeping quality of different parts of bluefin tuna.

## 2. Materials and Methods

### 2.1. Materials

The tuna used in the study was purchased from Dalian Chenyang Technology Development Co., Ltd. It was frozen immediately after fishing, cut according to the naked body, big belly and middle belly, and then it was sent to the laboratory for frozen storage.

### 2.2. Main Test Equipment

LF-NMR measurements were carried out by using a Niumag Benchtop Pulsed NMR Analyzer (Niumag Electric Corporation, Shanghai, China) to analyze the water distribution and migration. A color difference meter (CR-400, Konica Minolta, Tokyo, Japan), was used for measuring color changes in the sample. A UV-V spectrophotometer (UV-1102, Shanghai Tianmei Instrument Co., Ltd., Shanghai, China) was used for measuring FAA. High-speed centrifuge was used to centrifuge the mixed solutions. A fluorescence spectrophotometer was used for measuring the endogenous fluorescence of protein.

### 2.3. Experimental Method

Tuna samples were grouped according to the naked body, middle belly and big belly. Then, 6 parallel tests were conducted in one experiment, and the tuna samples were stored in refrigerators at −55 °C and −18 °C for 6 months. After the tuna arrived in the laboratory, the fresh, initial values including water-holding capacity, color difference, MDA, salt-soluble protein content and endogenous fluorescent proteins were measured and then the experiment was carried out every month.

### 2.4. Thawing Method

Tuna was thawed in warm salt-water with a concentration of 3% and the probe of the thermometer was inserted into the sample to measure the central temperature. When the temperature reached 5 °C, it was considered that the thawing was complete.

### 2.5. Water-Holding Capacity

After thawing the tuna meat, the water on the surface was wiped off with filter paper. The meat was weighed, and 2 g was wrapped with two layers of paper, and put in the centrifugal tube. The meat was centrifuged at 4 °C for 10 min, at 5500 R/min, and then the mass of the meat was accurately weighed after centrifugation. The formula for the water-holding capacity is as follows:

W (%) = (M_a_/M_b_) × 100% (1)

where W is the water-holding capacity, M_a_ is the quality of meat after centrifugation, and M_b_ is the quality of meat before centrifugation.

### 2.6. LF-NMR Measurements

The prepared tuna sample was packed with fresh-keeping film and put into a nuclear magnetic detection tube with a diameter of 70 mm. The coil temperature was 32 °C, and the proton resonance frequency was adjusted to 24 MHz. The CPMG sequence was selected and T2 measurement parameters was set as: sampling frequency SW = 100 kHz, analog gain RG1 = 20, P1 = 18.00 μs, digital gain DRG1 = 6, TD = 400 004, PRG = 1, repeat sampling interval TW = 2000 ms, accumulation times NS = 4, P2 = 34.00 μs, echo time TE = 0.550, and serial number of the echo = 8000. According to the CPMG exponential decay curve, the transverse relaxation time T2 map was obtained by iterative inversion with analysis software.

### 2.7. MDA

One gram of tuna meat was added to 9 mL of normal saline, and centrifuged (7550 R/min, 10 min), After centrifugation, the supernatant was determined by visible spectrophotometry and by using a malondialdehyde kit (Nanjing Jiancheng Bioengineering Institute, Nanjing, China). Each group of samples was measured three times and the average value was taken.

### 2.8. Salt-Soluble Protein Assay

Next, 20 mL of a 0.02 mol/L PBS solution was added to 2 g of chopped tuna. The resultant precipitate was then collected after the mixed solution had been homogenized at high speed (6000 R/min, 3 min) and centrifuged at a low temperature (10,000× *g*, 4 °C, 20 min). After this procedure, 40 mL NaCl solution was prepared. Then, we added 12 mL 3% NaCl solution, homogenized the mixture at a high speed (6000 R/min, 3 min), centrifuged the mixture at a low temperature (10,000× *g*, 4 °C, 10 min), took the supernatant for storage, repeated the experiment (a total of 3 times), and combined the supernatant. At this time, the supernatant contained myogenic protein, and then a 20 μL sarcoplasmic protein solution was drained into a well-plate (dry in advance). The above draining process was repeated 3 times and the solution was placed in 3 orifice plates, respectively, as a parallel experiment. Then, 200 μL of Bradford solution was added to these 3 orifice plates and was quickly mixed at a room temperature of 25–30 °C. After a 5 min reaction, the microplate reader was inserted and connected to the computer for testing.

### 2.9. FAA

After homogenizing, drying, and grinding the sample, 0.2 g of dry sample was placed in a 55 mL volumetric flask, and 0.02 mol/L hydrochloric acid was added to a constant volume; it stood for 1 h, and was then filtered, purified, and passed through the membrane. The AccQ-Tag method was used to develop a reverse phase separation, which was carried out with UV 248 nm detection, and the instrument used was a high performance liquid chromatograph.

### 2.10. Endogenous Fluorescent Protein

Two grams of tuna meat was weighed and 18 mL of distilled water was added, it was homogenized (14,000 R/min, duration 1 min), and then the solution was centrifuged for 10 min (10,000 R/min, 4 °C), filtered to obtain precipitation. Then, 18 mL of NaCl solution with 3% concentration was added, it was homogenized, centrifuged and the supernatant was taken.

The extracted myofibrin solution was diluted to 0.05 mg/mL in 0.6 mol/L KCL solution, and it was set according to the following parameters: laser wavelength 295 nm, excitation, and emission slit width 2.5 nm. The wavelength scanning surface was divided into 300–400 nm, the scanning speed was 12,000 nm/min, and each sample was measured in parallel 5 times.

### 2.11. Color Difference

The thawed tuna was cut into 20 mm × 20 mm × 15 mm, a CR-400 color difference meter was used to measure the L*, a* value, and ΔE of the tuna block, and whiteboard correction was finished before sample determination. Each group of samples was measured in parallel 3 times.

### 2.12. Data Analysis

The data for all indexes were processed by Excel 2016. The measured indexes were measured three times with parallel measurement results, drawn by origin 8.5 software (OriginLab, Northampton, MA, USA), and analyzed by SPSS 19.0 software (SPSS Inc., Chicago, IL, USA) (*p* < 0.05 is considered a significant difference).

## 3. Results

### 3.1. Changes of Muscle Water-Holding Capacity in Different Parts of Tuna

**Figure 1:**
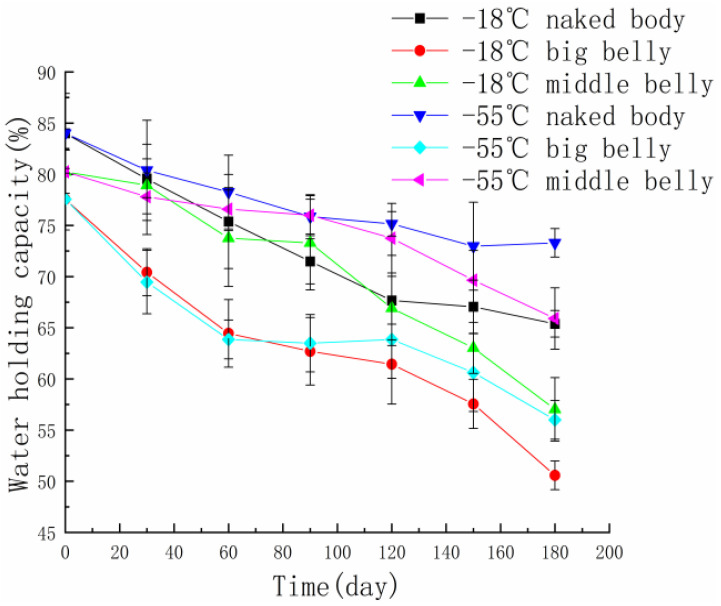
The change in water-holding capacity in different parts of tuna during frozen storage at different temperatures.

The water-holding capacity is defined as the physical binding force of a tuna sample to water under the action of external forces [14]. Water is the main component of tuna muscle, and water content has an important impact on the taste and quality of tuna muscle [15]. The three-dimensional network structure in the muscle can affect the distribution state and form of water, so the water-holding capacity of tuna meat is also affected by its muscle composition [16]. Figure 1 shows the change in water-holding capacity of tuna sampled from different parts when frozen at −18 °C and −55 °C. It can be seen that during frozen storage time, the water-holding capacity of tuna meat shows a downward trend, and the water-holding capacity of tuna meat stored at −18 °C is relatively lower than that frozen at −55 °C. At the same temperature, the water-holding capacity of the naked body tuna is stronger, and the water-holding capacity of the belly is weaker because the water-holding capacity of the tuna meat is closely related to the content of protein. The water in tuna meat is in the form of free water. One part of the free water exists between myofibrils and connective tissue, and the other part is combined with the carboxyl groups of proteins and sugars. In the process of frozen storage, the hydrophobic/hydrophilic binding bond around the protein is destroyed, and the water bound with the protein becomes free water, resulting in a decrease in the water-holding capacity [17]. In addition, the formation of ice crystals causes certain damage to the tissue structure of myofibrils, which is also an important factor affecting the water-holding capacity of tuna. The size of the ice-crystal was also affected by the freezing temperature; freezing at lower temperatures resulted in smaller ice crystals and less modification of the food’s solid structures [18]. The water-holding capacity of the tuna samples from different parts was better at the frozen temperature of −55 °C.

### 3.2. Changes in Muscle Color in Different Parts of Tuna

**Figure 2:**
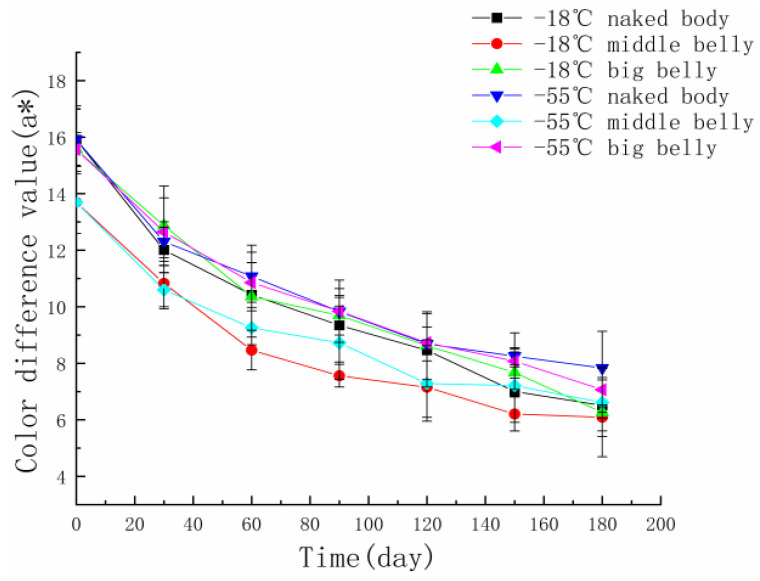
The color change in different parts of tuna during frozen storage at different temperatures.

Biochemical changes such as protein oxidation and fat oxidation in tuna meat will change the color. This is an important index to evaluate the quality of tuna. The color of tuna meat will have an intuitive impact on consumers’ purchases [19]. Fresh tuna is bright red, and the color of the meat is expressed by the value of a*. The change in the color of tuna meat is related to the formation of high-iron myoglobin. During frozen storage, ferrous ions bound to myoglobin are easily oxidized to high-iron myoglobin, which results in browning of the flesh [20]. Figure 2 shows that the value of the color difference of the tuna meat had a downward trend over the whole storage period. In the early stage of storage, the a* value decreased rapidly, a large amount of high-iron myoglobin was produced, and the meat color became significantly darker. In the later stage of storage, the decrease in a* slowed down and gradually stabilized. The protein content in the naked body part of tuna is high. During storage, ferrous ions are further oxidized to produce high-iron myoglobin, resulting in the rapid decline in the a* value. Tuna’s big belly contains less protein, but it contains more fat, which can prevent tuna from having a bright red color. In addition, during the oxidation process, fats can produce free radicals that oxidize myoglobin and ferrous ions, producing high-iron myoglobin [21]. At the same temperature, the a* value of the big belly part of tuna is always lower than that of the middle belly and naked body part. After 180 days of storage, the naked body part of tuna at the storage temperature of −55 °C had the best a* value.

### 3.3. Changes in Muscle MDA in Different Parts of Tuna

**Figure 3:**
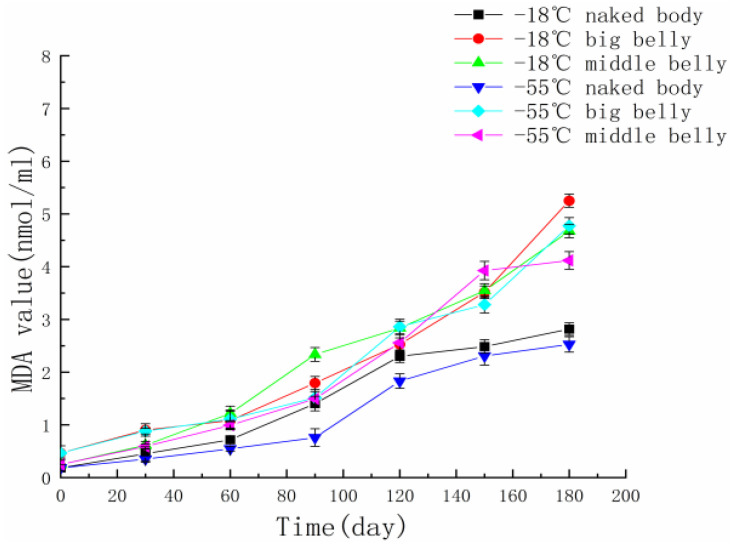
MDA changes in different parts of tuna during frozen storage at different temperatures.

In the whole process of frozen storage, fat oxidation leads to bad-smelling tuna meat, and the deterioration of the tuna meat, which affects its quality and flavor and reduces the commercial value of the meat. After oxidative degradation, unsaturated fatty acids produce malondialdehyde. The measurement of malondialdehyde can determine the degree of oxidation of tuna fat and characterize the freshness of aquatic products [22]. As shown in Figure 3, during the frozen storage of bluefin tuna naked body, big belly and middle belly at −18 °C and −55 °C, the MDA content continued to rise, which is due to the continuous accumulation of fat oxidation products with the extension of frozen storage time. The content of MDA is significantly affected by temperature and location, and the same location is frozen at different temperatures. The lower the temperature, the slower the increase in MDA content, which is because the low-temperature environment can slow down the reaction rate and reduce the formation of aldehydes, ketones, and carboxylic acids. Under the same temperature, the MDA content of the naked body part was significantly lower than that of the big belly and middle belly, which was consistent with the fat content of the three parts of the tuna. Xu et al. [23] studied the lipid and myoglobin oxidation of bluefin tuna frozen at different temperatures, and obtained similar results.

### 3.4. Changes in Salt-Soluble Protein Content in Different Parts of Tuna Muscle

**Figure 4:**
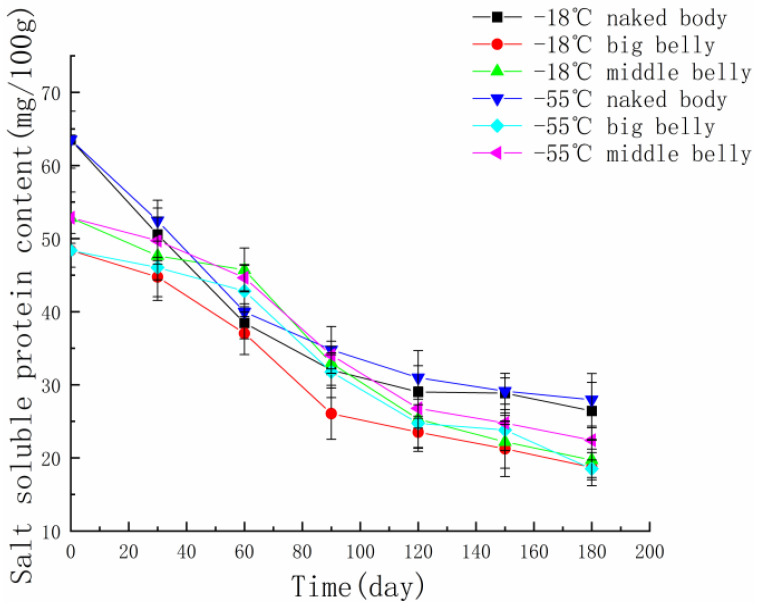
The changes in salt-soluble protein content in different parts of tuna during frozen storage at different temperatures.

Protein is the main component of tuna meat. The content of salt-soluble protein in muscle protein accounts for more than 60%. The content of salt-soluble protein can reflect the degree of tuna denaturation [24]. Figure 4 shows the changes in the protein content of tuna meat in different parts at different temperatures. During the process of frozen storage, the content of salt-soluble protein decreased significantly, and the decline rate gradually slowed down, which is due to the over-aggregation and denaturation of hydrogen bonds, disulfide bonds, and hydrophobic bonds in myofibrillar protein molecules [25]. The level of frozen storage temperature has an impact on the content of salt-soluble protein. In the early stage of frozen storage, the protein content of tuna meat frozen at −18 °C decreases rapidly, and the salt-soluble protein content of tuna meat frozen at −55 °C decreases slowly because the low temperature can inhibit the activity of enzymes in organisms. At the end of frozen storage, there is no significant difference in the content of salt-soluble protein of tuna meat frozen at different temperatures, which may be because the mechanical action of ice crystal destroys the myofibril structure, and the protein becomes alkali-soluble protein after freeze denaturation, resulting in the decrease in salt-soluble protein content [26]. After comparing the protein content of three different parts of tuna meat, it was found that the content of salt-soluble protein in the naked body part of tuna meat is the highest and that in the big belly is the lowest, which is due to the different protein content in muscle in different parts of tuna.

### 3.5. FAA Content in Different Parts of Tuna Muscle

There are many kinds of amino acids in food, mainly including FAAs and bound amino acids. Different kinds of amino acids show different flavors [27]. The composition and content of amino acids in food determine the flavor of food. FAAs come from protein hydrolysis. Aspartic acid, serine, glutamic acid, glycine, alanine, and proline are amino acids that provide flavor, while threonine, valine, methionine, isoleucine, leucine, phenylalanine, and lysine are essential amino acids, which supply human nutrition [28]. Table 1 shows the content of FAAs in different parts of tuna after 180 days of storage at different temperatures. It can be seen that after frozen storage, the FAA content of tuna frozen at −18 °C in the same part is significantly lower than that frozen at −55 °C, which is consistent with the determination results of salt-soluble protein, indicating that reducing the frozen storage temperature can reduce the degree of protein degradation and the content of FAA. At the same temperature, the content of amino acids in the middle belly of tuna is the highest, and the total content of amino acids in the naked body part is lower. Glycine, histidine, glutamic acid, etc., are odorous amino acids, and the increase in odorous amino acid content will lead to a certain bitterness in tuna, leading to the decline in taste [29]. When frozen at the same temperature, the content of odor acid in naked body parts was the lowest, and the change in the flavor of tuna was the least during frozen storage.

### 3.6. Endogenous Fluorescent Proteins in Different Parts of Tuna Muscle

**Figure 5:**
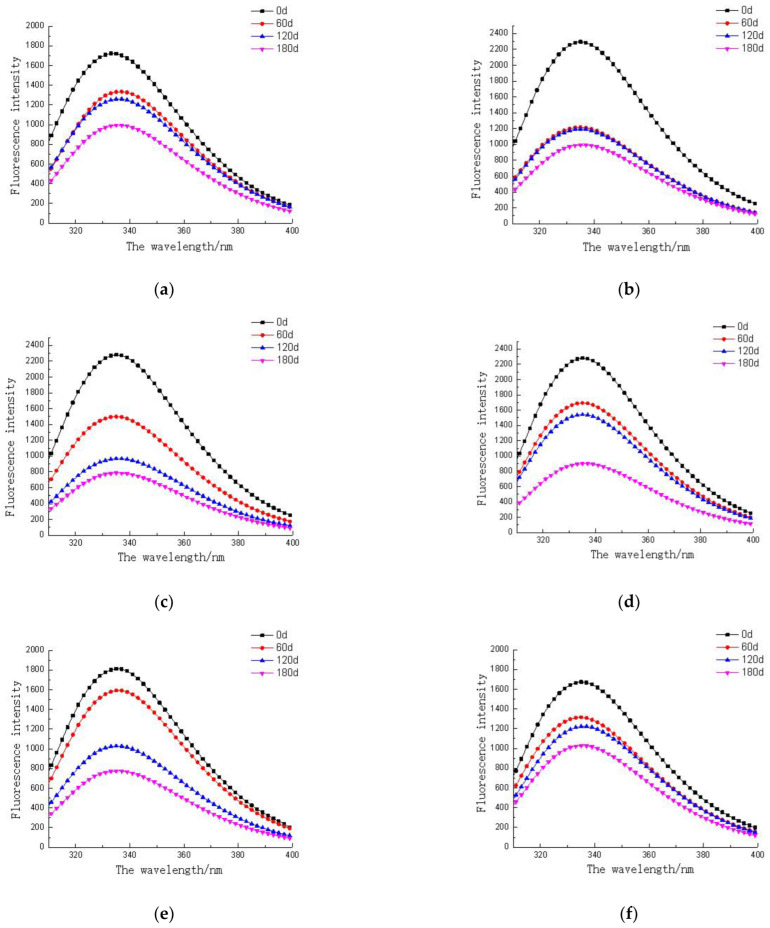
Changes in endogenous fluorescence intensity of tuna in different parts during frozen storage at different temperatures (**a**) frozen storage at −18 °C (**b**) frozen storage at −18 °C (**c**) frozen storage at −18 °C (**d**) frozen storage at −55 °C (**e**) frozen storage at −18 °C (**f**) frozen storage at −55 °C).

The internal fluorescence of protein reflects the changes in the tertiary structure of protein molecules. It contains tryptophan, tyrosine, and phenylalanine residues, and can produce fluorescence under the excitation light of 280 nm or 295 nm. By detecting the change in the tryptophan residue microenvironment, the internal structure change in protein can be obtained, and the change in the quality of tuna meat can be determined [30]. Figure 5 shows the changes in endogenous protein fluorescence in different parts of tuna frozen at different temperatures. With the extension of frozen storage time, the endogenous fluorescence intensity of muscle showed a downward trend. This is because low temperature destroys the molecular structure of the myofibrillar protein, exposes tryptophan residues and active groups of protein molecules, and intermolecular aggregation lead to tryptophan residues embedded in protein molecules, resulting in the decrease in endogenous fluorescence intensity [31]. Tuna meat in the same part was frozen at −55 °C, and the decline was small, indicating that the temperature can slow down the degree of protein oxidation and prevent the destruction of protein molecular structure. The order of endogenous fluorescence intensity of tuna frozen at the same temperature is as follows: naked body part > middle belly part > big belly part, which is consistent with the protein content of tuna in different parts.

### 3.7. Water Distribution and Migration in Different Parts of Tuna Muscle

**Figure 6:**
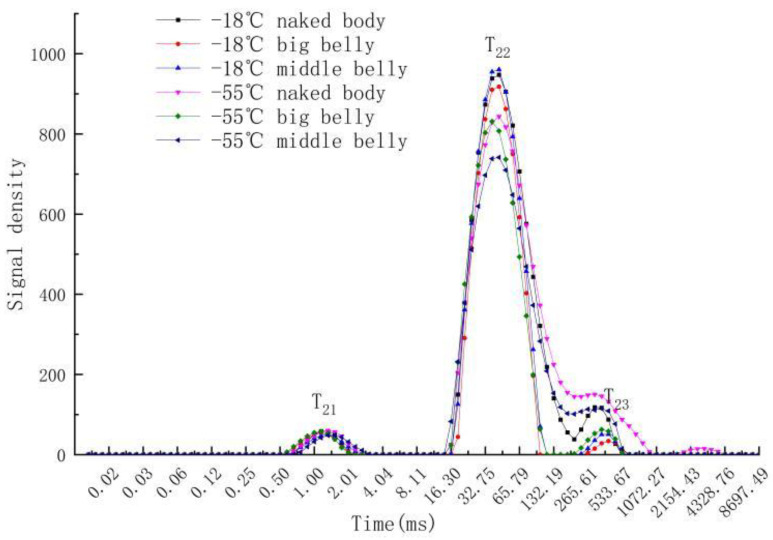
Changes in lateral relaxation time in different parts of tuna under different temperatures.

LF-NMR uses ^1^H proton as the medium to determine the water content in the sample by measuring the processing time (relaxation time) of ^1^H proton from a high-energy state to a low-energy state [32]. The transverse relaxation time is represented by T_2_, and the distribution state of T_2_ represents the distribution of water, indicating the degree of freedom of water molecules. T_21_ (1~10 ms) represents the combination of water and macromolecules in the form of bound water. T_22_ (10–155 ms) represents water present in the myofibril network and T_23_ (155 ms~) represents water that can flow freely [33]. The changes in the relaxation time of tuna in different parts at different frozen storage temperatures is shown in Figure 6. The peak area of the curve represents the distribution of the water state. There is little difference in the peak area of tuna in all frozen storage experimental groups within 0–10 MS, which indicates that different frozen storage temperatures have little effect on the bound water of tuna in different parts. The water content of T_22_ frozen at −55 °C is significantly higher than that frozen at −18 °C; Harnkarnsujarit et al. [34] obtained a similar result. For frozen tuna, the content of bound water and free water is closely related to the frozen storage temperature; a lower storage temperature results in a lower portion of free water.

Table 2 shows the changes in water in different states during the frozen storage time of tuna. The internal water of tuna mainly exists in the form of immobilized water, which accounts for more than 90% of the total water content [35]. For tuna meat frozen at the same temperature, the water content in the naked body part was the highest, and that in the big belly was the lowest. This is because the fat content in the big belly is high, and the tissue structure is fragile. During the process of frozen storage, the water-holding capacity of the myofibril network decreases, and it is not easy to convert immobilized water into free water, resulting in a large loss of water. After 180 days of frozen storage, the naked body part of tuna stored at −55 °C has the highest water content, of which the proportion of immobilized water was as high as 95.7%, indicating that the myofibril structure of tuna was best preserved. Similar studies have also found that the stability of protein structures highly influences the water-holding capacity of seafoods and a higher degree of protein aggregations and denaturation causes low water-holding capacity [36].

**Figure 7:**
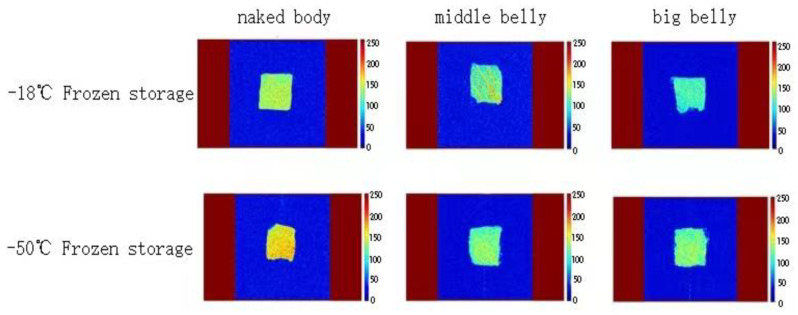
The pseudo color images of different parts of tuna frozen at different temperatures.

LF-NMR can generate pseudo color graphics to display the distribution of water. The stronger the brightness in the image, the higher the signal intensity in this part and the higher the water content in the sample [37]. Figure 7 shows the water distribution of different parts of tuna frozen at different temperatures. The effect of frozen storage temperature on the water distribution of tuna meat is relatively small, and the water distribution in different parts is significantly different. In the naked body part frozen at −55 °C, the tuna meat pattern shows red and yellow, which is evenly distributed, indicating that the signal intensity is high and the water content is the highest. The color of the middle belly and big belly tuna is dim and unevenly distributed, which is due to the high content of fat in the middle belly and big belly, and the distribution of fat affects the water distribution and migration.

The tuna was better maintained after 180 days of frozen storage temperature at −55 °C but the ice crystals formed by frozen storage can damage the salt-soluble protein in the tuna and decrease the hardness of the tuna. Although the shelf life was prolonged, the hardness and taste of tuna meat decreased. Liaomingtao et al. [38] studied the influence of frozen storage temperature on the color change in tuna meat. The quality of the naked body of tuna was better than the quality of the middle belly and big belly parts of tuna when they were stored frozen at −18 °C and −55 °C. The MDA value increased when the storage time increased. The higher the fat content, the more the change in the quality of the tuna; but, for short-term storage for sales, −18 °C was a suitable frozen storage temperature.

## 4. Conclusions

In this paper, the effects of frozen storage temperatures of −18 °C and −55 °C on the water-holding capacity and physicochemical properties of muscles in different parts of bluefin tuna were studied. The results showed that the water-holding capacity, salt-soluble protein, and water content of tuna decreased as the storage time increased. Compared to the frozen storage temperature of −18 °C, better-quality bluefin tuna was obtained at the frozen storage temperature of −55 °C. Because there was little change in the quality during short-term frozen storage, the frozen storage temperature of −18 °C can be suitable for short-term frozen storage for sales. The protein content and the content of salt-soluble protein was highest in the naked body part. There is more fat in the big belly and middle belly, which leads to a rapid rise in MDA in the other two parts. The amino acid content in the middle belly was the highest, and the odor and amino acid content in the naked body part was the lowest. After 180 days of storage, the naked body part of tuna at the storage temperature of −55 °C has the best a* value, the highest water content and the best water distribution. The results can help to enhance the economic value of bluefin tuna sales. In future research, the big belly and the middle belly of the bluefin tuna in frozen storage requires more attention, and the relationship between the quality of the tuna and the energy consumption of the frozen storage should be considered.

## Figures and Tables

**Table 1:** Contents of FAAs in different parts of tuna frozen at different temperatures.

Type	−18 °C Naked Body	−18 °C Middle Belly	−18 °C Big Belly	−55 °C Naked Body	−55 °C Middle Belly	−55 °C Big Belly
Aspartic acid	8.45278	28.5659	15.1902	7.82438	11.8069	9.36199
threonine	26.9676	62.6204	42.3936	26.1867	28.8607	24.9122
serine	6.08948	28.302	9.33835	6.92564	10.4408	7.08034
glutamate	29.402	77.3983	56.1695	27.7802	42.5536	44.1216
glycine	43.6959	98.0136	61.2816	39.8779	61.3309	36.0496
alanine	143.353	264.492	108.758	135.117	109.865	73.713
valine	36.3519	69.6088	47.6301	35.8909	32.9729	27.891
methionine	9.01322	38.6347	29.3612	10.7306	9.09695	16.0305
isoleucine	20.7363	36.0292	22.5213	21.1904	15.4014	13.831
leucine	35.9597	61.8645	39.3984	35.2674	27.7481	24.0869
Tyrosine	24.8361	54.2872	32.2359	23.0676	28.4776	21.9596
Phenylalanine	16.8455	45.6567	20.1593	14.4584	19.2527	10.7646
Lysine	264.279	1310.84	141.166	242.389	523.28	98.1079
histidine	8339.41	8802.48	9119.27	7127.83	9926.31	8101.62
Arginine	53.4648	36.7473	32.9904	44.2048	60.5516	20.2973
total	9058.857	11,015.54	9777.864	7798.741	10,907.85	8529.828

**Table 2:** Proportion of frozen storage peak area in different parts of tuna at different temperatures.

Frozen Storage Mode	Total Peak Area (Water Content)	Bound Water (T21, %)	Immobilized Water (T22, %)	Free Water (T23, %)
−18 °C big belly	7455.455	0.041843	0.941708	0.016449
−18 °C middle belly	8228.852	0.035899	0.941465	0.022635
−18 °C naked body	9767.239	0.032556	0.91532	0.052174
−55 °C big belly	7559.581	0.044403	0.919212	0.036385
−55 °C middle belly	8683.41	0.035597	0.890377	0.074526
−55 °C naked body	10,452.81	0.035959	0.957319	0.006723

## Data Availability

Authors can confirm that all relevant data are included in the article.
